# Childhood cancer survivors in India: gender gap in long-term survivorship care

**DOI:** 10.1016/j.lansea.2026.100851

**Published:** 2026-08-26

**Authors:** Maya Prasad

**Affiliations:** Paediatric Oncology, Tata Memorial Centre, Mumbai, India

Although childhood cancer survival rates are increasing in India, survivorship support systems remain limited, particularly for girls and young women.^1^^,^^2^ Robust sex-disaggregated survivorship data from low-income and middle-income countries remain scarce, and survivorship programmes are generally challenged by fragmented long-term follow-up, challenges in transition to adult care, workforce limitations, and attrition from survivorship services.^3^ Gender disparity begins early in the cancer journey, with delayed diagnosis, unequal access to care, and higher treatment abandonment among girls in India compared to boys.^2^ As survival improves, the long-term health and social outcomes of women survivors are becoming increasingly important.

The burden of survivorship is not uniform. Women survivors experience reproductive, sexual and endocrine issues, including late effects such as infertility, premature ovarian insufficiency, adverse pregnancy outcomes, and psychological distress.^3^, ^4^, ^5^ In countries of Southeast Asia, these late effects are often compounded by prevailing gender inequities, including limited education and employment opportunities, financial dependence, and restricted access to survivorship care. Though similar reproductive, psychosocial, and transition-related concerns are seen in men, these complications may have greater social consequences for women because of prevailing expectations around marriage and childbearing.^3^

These consequences become more apparent with increasing age over long term follow up. While most girls affected by childhood cancer complete therapy, fewer women remain in follow-up as they move into adulthood. Marriage, relocation, fragmented transition services, financial dependence, and caregiving responsibilities may contribute to attrition.^2^^,^^3^ This is concerning because many endocrine, reproductive, cardiovascular, and metabolic late effects become clinically relevant only during early adult life.^2^, ^3^, ^4^, ^5^ Survivors may require long-term breast surveillance after chest radiation, cervical cancer screening, nutritional rehabilitation, and counselling regarding pregnancy-related cardiovascular risk.^3^, ^4^, ^5^ Our observation of progressive attrition is consistent with the broader recognition that survivorship outcomes are influenced not only by treatment-related late effects but also by health-system and sociocultural factors. Progressive disengagement from long-term follow-up should be considered a gender-responsive survivorship outcome.

Optimising reproductive health of women survivors requires structured counselling regarding fertility and other sexual and reproductive health needs (Table 1).^3^, ^4^, ^5^ Where feasible, fertility preservation counselling should begin before treatment and should include access to multidisciplinary services. They should receive optimised clinical care integrating oncologists, physicians, gynaecologists, reproductive endocrinologists, fertility specialists, psychologists, and primary care providers.^3^^,^^5^ At the health-system level, structured transition pathways, risk-stratified survivorship clinics, shared-care models, survivorship registries, routine sex-disaggregated outcome reporting, quality of life assessments, provider education, and national survivorship guidelines should be prioritised to improve long-term retention in care and reduce gender-related disparities in survivorship outcomes.^3^^,^^5^ School re-entry, vocational rehabilitation, and transition into higher education and employment must also be prioritised for young women.^2^^,^^3^^,^^5^ As survival improves, equitable, gender-responsive survivorship care should become a core component of national childhood cancer control programmes and universal health coverage.

**Table 1:** Key gender-specific survivorship priorities among women surviving childhood cancer in India.

Domain	Key priorities
Reproductive survivorship	Infertility, fertility preservation, menstrual dysfunction, premature ovarian insufficiency, sexual health, pregnancy planning, and multidisciplinary reproductive care.
Psychosocial survivorship	Stigma, concealment of cancer history, concerns regarding infertility and marriageability, body image concerns, interrupted education, and challenges with school re-entry and social reintegration
Transition and long-term follow-up	Progressive disengagement from survivorship care during adulthood, particularly after transition from paediatric care, marriage, and relocation
Preventive and supportive survivorship	HPV vaccination, breast and cervical cancer surveillance, anaemia management, nutritional rehabilitation, and long-term health promotion
Health-system challenges	Structured transition pathways, risk-stratified survivorship clinics, shared-care models, survivorship registries, provider education, and national survivorship guidelines.

## Contributors

MP conceptualised the manuscript, reviewed the literature, and wrote the manuscript.

## Statement on AI use

During the preparation of this work, we used Grammarly AI to assist with grammar checking and language refinement. After using this tool, the author reviewed and edited the content as needed and take full responsibility for the final content of the publication.

## Declaration of interests

The author declares no conflicts of interest.
